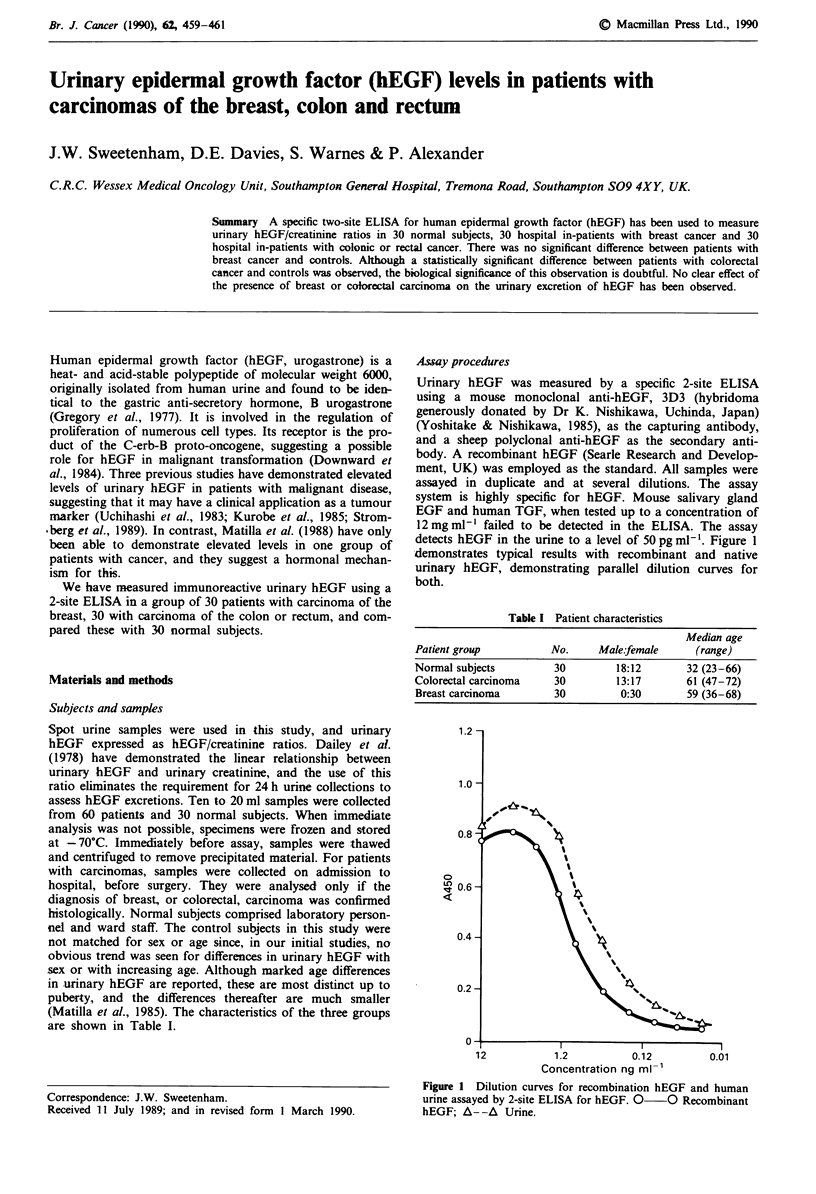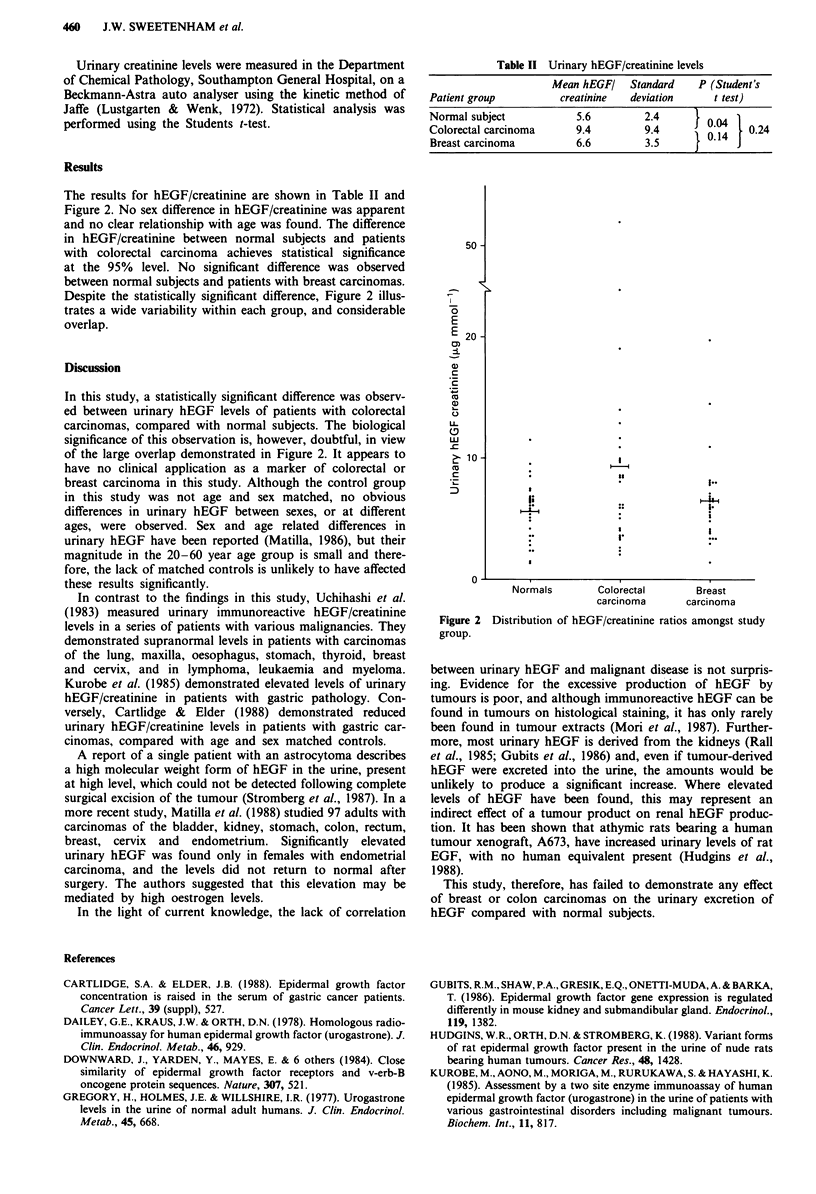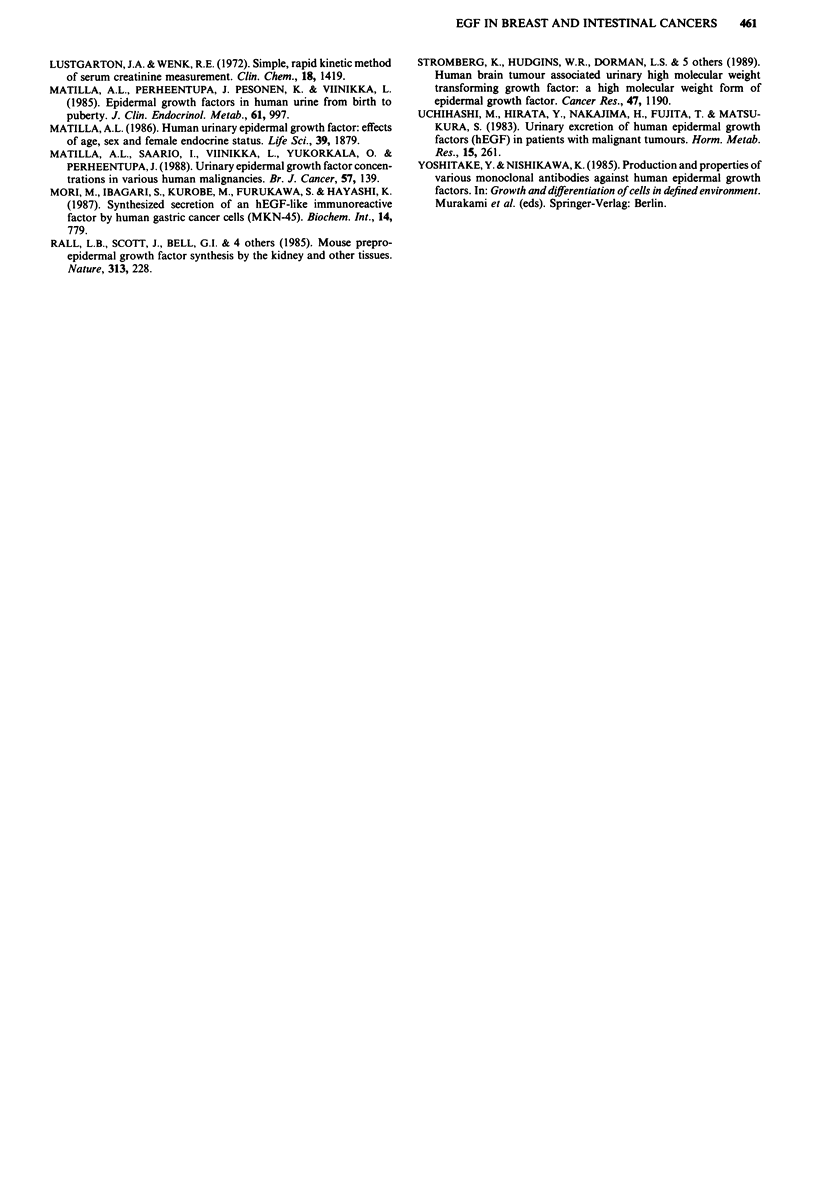# Urinary epidermal growth factor (hEGF) levels in patients with carcinomas of the breast, colon and rectum.

**DOI:** 10.1038/bjc.1990.318

**Published:** 1990-09

**Authors:** J. W. Sweetenham, D. E. Davies, S. Warnes, P. Alexander

**Affiliations:** C.R.C. Wessex Medical Oncology Unit, Southampton General Hospital, UK.

## Abstract

A specific two-site ELISA for human epidermal growth factor (hEGF) has been used to measure urinary hEGF/creatinine ratios in 30 normal subjects, 30 hospital in-patients with breast cancer and 30 hospital in-patients with colonic or rectal cancer. There was no significant difference between patients with breast cancer and controls. Although a statistically significant difference between patients with colorectal cancer and controls was observed, the biological significance of this observation is doubtful. No clear effect of the presence of breast or colorectal carcinoma on the urinary excretion of hEGF has been observed.


Br. J. Cancer (1990), 62, 459-461 © Macmillan Press Ltd., 1990

### Urinary epidermal growth factor (hEGF) levels in patients with carcinomas of the breast, colon and rectum

J.W. Sweetenham, D.E. Davies, S. Warnes & P. Alexander

*C.R.C. Wessex Medical Oncology Unit, Southampton General Hospital, Tremona Road, Southampton SO9 4XY, UK.*

**Summary** A specific two-site ELISA for human epidermal growth factor (hEGF) has been used to measure urinary hEGF/creatinine ratios in 30 normal subjects, 30 hospital in-patients with breast cancer and 30 hospital in-patients with colonic or rectal cancer. There was no significant difference between patients with breast cancer and controls. Although a statistically significant difference between patients with colorectal cancer and controls was observed, the biological significance of this observation is doubtful. No clear effect of the presence of breast or colorectal carcinoma on the urinary excretion of hEGF has been observed.

Human epidermal growth factor (hEGF, urogastrone) is a heat- and acid-stable polypeptide of molecular weight 6000, originally isolated from human urine and found to be identical to the gastric anti-secretory hormone, B urogastrone (Gregory *et al.*, 1977). It is involved in the regulation of proliferation of numerous cell types. Its receptor is the product of the C-erb-B proto-oncogene, suggesting a possible role for hEGF in malignant transformation (Downward *et al.*, 1984). Three previous studies have demonstrated elevated levels of urinary hEGF in patients with malignant disease, suggesting that it may have a clinical application as a tumour marker (Uchihashi *et al.*, 1983; Kurobe *et al.*, 1985; Stromberg *et al.*, 1989). In contrast, Matilla *et al.* (1988) have only been able to demonstrate elevated levels in one group of patients with cancer, and they suggest a hormonal mechanism for this.

We have measured immunoreactive urinary hEGF using a 2-site ELISA in a group of 30 patients with carcinoma of the breast, 30 with carcinoma of the colon or rectum, and compared these with 30 normal subjects.

### Materials and methods

### *Subjects and samples*

Spot urine samples were used in this study, and urinary hEGF expressed as hEGF/creatinine ratios. Dailey *et al.* (1978) have demonstrated the linear relationship between urinary hEGF and urinary creatinine, and the use of this ratio eliminates the requirement for 24 h urine collections to assess hEGF excretions. Ten to 20 ml samples were collected from 60 patients and 30 normal subjects. When immediate analysis was not possible, specimens were frozen and stored at −70°C. Immediately before assay, samples were thawed and centrifuged to remove precipitated material. For patients with carcinomas, samples were collected on admission to hospital, before surgery. They were analysed only if the diagnosis of breast, or colorectal, carcinoma was confirmed histologically. Normal subjects comprised laboratory personnel and ward staff. The control subjects in this study were not matched for sex or age since, in our initial studies, no obvious trend was seen for differences in urinary hEGF with sex or with increasing age. Although marked age differences in urinary hEGF are reported, these are most distinct up to puberty, and the differences thereafter are much smaller (Matilla *et al.*, 1985). The characteristics of the three groups are shown in Table I.

### *Assay procedures*

Urinary hEGF was measured by a specific 2-site ELISA using a mouse monoclonal anti-hEGF, 3D3 (hybridoma generously donated by Dr K. Nishikawa, Uchinda, Japan) (Yoshitake & Nishikawa, 1985), as the capturing antibody, and a sheep polyclonal anti-hEGF as the secondary antibody. A recombinant hEGF (Searle Research and Development, UK) was employed as the standard. All samples were assayed in duplicate and at several dilutions. The assay system is highly specific for hEGF. Mouse salivary gland EGF and human TGF, when tested up to a concentration of $12\ mg\ ml^{-1}$ failed to be detected in the ELISA. The assay detects hEGF in the urine to a level of $50\ pg\ ml^{-1}$. Figure 1 demonstrates typical results with recombinant and native urinary hEGF, demonstrating parallel dilution curves for both.

**Table I** Patient characteristics

| *Patient group* | *No.* | *Male:female* | *Median age (range)* |
|---|---|---|---|
| Normal subjects | 30 | 18:12 | 32 (23-66) |
| Colorectal carcinoma | 30 | 13:17 | 61 (47-72) |
| Breast carcinoma | 30 | 0:30 | 59 (36-68) |

**Figure 1** Dilution curves for recombination hEGF and human urine assayed by 2-site ELISA for hEGF. O——O Recombinant hEGF; Δ--Δ Urine.

Correspondence: J.W. Sweetenham.
Received 11 July 1989; and in revised form 1 March 1990.

Urinary creatinine levels were measured in the Department of Chemical Pathology, Southampton General Hospital, on a Beckmann-Astra auto analyser using the kinetic method of Jaffe (Lustgarten & Wenk, 1972). Statistical analysis was performed using the Students *t*-test.

### Results

The results for hEGF/creatinine are shown in Table II and Figure 2. No sex difference in hEGF/creatinine was apparent and no clear relationship with age was found. The difference in hEGF/creatinine between normal subjects and patients with colorectal carcinoma achieves statistical significance at the 95% level. No significant difference was observed between normal subjects and patients with breast carcinomas. Despite the statistically significant difference, Figure 2 illustrates a wide variability within each group, and considerable overlap.

### Discussion

In this study, a statistically significant difference was observed between urinary hEGF levels of patients with colorectal carcinomas, compared with normal subjects. The biological significance of this observation is, however, doubtful, in view of the large overlap demonstrated in Figure 2. It appears to have no clinical application as a marker of colorectal or breast carcinoma in this study. Although the control group in this study was not age and sex matched, no obvious differences in urinary hEGF between sexes, or at different ages, were observed. Sex and age related differences in urinary hEGF have been reported (Matilla, 1986), but their magnitude in the 20–60 year age group is small and therefore, the lack of matched controls is unlikely to have affected these results significantly.

In contrast to the findings in this study, Uchihashi *et al.* (1983) measured urinary immunoreactive hEGF/creatinine levels in a series of patients with various malignancies. They demonstrated supranormal levels in patients with carcinomas of the lung, maxilla, oesophagus, stomach, thyroid, breast and cervix, and in lymphoma, leukaemia and myeloma. Kurobe *et al.* (1985) demonstrated elevated levels of urinary hEGF/creatinine in patients with gastric pathology. Conversely, Cartlidge & Elder (1988) demonstrated reduced urinary hEGF/creatinine levels in patients with gastric carcinomas, compared with age and sex matched controls.

A report of a single patient with an astrocytoma describes a high molecular weight form of hEGF in the urine, present at high level, which could not be detected following complete surgical excision of the tumour (Stromberg *et al.*, 1987). In a more recent study, Matilla *et al.* (1988) studied 97 adults with carcinomas of the bladder, kidney, stomach, colon, rectum, breast, cervix and endometrium. Significantly elevated urinary hEGF was found only in females with endometrial carcinoma, and the levels did not return to normal after surgery. The authors suggested that this elevation may be mediated by high oestrogen levels.

In the light of current knowledge, the lack of correlation between urinary hEGF and malignant disease is not surprising. Evidence for the excessive production of hEGF by tumours is poor, and although immunoreactive hEGF can be found in tumours on histological staining, it has only rarely been found in tumour extracts (Mori *et al.*, 1987). Furthermore, most urinary hEGF is derived from the kidneys (Rall *et al.*, 1985; Gubits *et al.*, 1986) and, even if tumour-derived hEGF were excreted into the urine, the amounts would be unlikely to produce a significant increase. Where elevated levels of hEGF have been found, this may represent an indirect effect of a tumour product on renal hEGF production. It has been shown that athymic rats bearing a human tumour xenograft, A673, have increased urinary levels of rat EGF, with no human equivalent present (Hudgins *et al.*, 1988).

This study, therefore, has failed to demonstrate any effect of breast or colon carcinomas on the urinary excretion of hEGF compared with normal subjects.

**Table II** Urinary hEGF/creatinine levels

| *Patient group* | *Mean hEGF/ creatinine* | *Standard deviation* | *P (Student's t test)* | |
|---|---|---|---|---|
| Normal subject | 5.6 | 2.4 | 0.04 | |
| Colorectal carcinoma | 9.4 | 9.4 | 0.14 | 0.24 |
| Breast carcinoma | 6.6 | 3.5 | | |

**Figure 2** Distribution of hEGF/creatinine ratios amongst study group.

### References

CARTLIDGE, S.A. & ELDER, J.B. (1988). Epidermal growth factor concentration is raised in the serum of gastric cancer patients. *Cancer Lett.*, **39** (suppl), 527.

DAILEY, G.E., KRAUS, J.W. & ORTH, D.N. (1978). Homologous radioimmunoassay for human epidermal growth factor (urogastrone). *J. Clin. Endocrinol. Metab.*, **46**, 929.

DOWNWARD, J., YARDEN, Y., MAYES, E. & 6 others (1984). Close similarity of epidermal growth factor receptors and v-erb-B oncogene protein sequences. *Nature*, **307**, 521.

GREGORY, H., HOLMES, J.E. & WILLSHIRE, I.R. (1977). Urogastrone levels in the urine of normal adult humans. *J. Clin. Endocrinol. Metab.*, **45**, 668.

GUBITS, R.M., SHAW, P.A., GRESIK, E.Q., ONETTI-MUDA, A. & BARKA, T. (1986). Epidermal growth factor gene expression is regulated differently in mouse kidney and submandibular gland. *Endocrinol.*, **119**, 1382.

HUDGINS, W.R., ORTH, D.N. & STROMBERG, K. (1988). Variant forms of rat epidermal growth factor present in the urine of nude rats bearing human tumours. *Cancer Res.*, **48**, 1428.

KUROBE, M., AONO, M., MORIGA, M., RURUKAWA, S. & HAYASHI, K. (1985). Assessment by a two site enzyme immunoassay of human epidermal growth factor (urogastrone) in the urine of patients with various gastrointestinal disorders including malignant tumours. *Biochem. Int.*, **11**, 817.

LUSTGARTON, J.A. & WENK, R.E. (1972). Simple, rapid kinetic method of serum creatinine measurement. *Clin. Chem.*, **18**, 1419.

MATILLA, A.L., PERHEENTUPA, J. PESONEN, K. & VIINIKKA, L. (1985). Epidermal growth factors in human urine from birth to puberty. *J. Clin. Endocrinol. Metab.*, **61**, 997.

MATILLA, A.L. (1986). Human urinary epidermal growth factor: effects of age, sex and female endocrine status. *Life Sci.*, **39**, 1879.

MATILLA, A.L., SAARIO, I., VIINIKKA, L., YUKORKALA, O. & PERHEENTUPA, J. (1988). Urinary epidermal growth factor concentrations in various human malignancies. *Br. J. Cancer*, **57**, 139.

MORI, M., IBAGARI, S., KUROBE, M., FURUKAWA, S. & HAYASHI, K. (1987). Synthesized secretion of an hEGF-like immunoreactive factor by human gastric cancer cells (MKN-45). *Biochem. Int.*, **14**, 779.

RALL, L.B., SCOTT, J., BELL, G.I. & 4 others (1985). Mouse prepro-epidermal growth factor synthesis by the kidney and other tissues. *Nature*, **313**, 228.

STROMBERG, K., HUDGINS, W.R., DORMAN, L.S. & 5 others (1989). Human brain tumour associated urinary high molecular weight transforming growth factor: a high molecular weight form of epidermal growth factor. *Cancer Res.*, **47**, 1190.

UCHIHASHI, M., HIRATA, Y., NAKAJIMA, H., FUJITA, T. & MATSUKURA, S. (1983). Urinary excretion of human epidermal growth factors (hEGF) in patients with malignant tumours. *Horm. Metab. Res.*, **15**, 261.

YOSHITAKE, Y. & NISHIKAWA, K. (1985). Production and properties of various monoclonal antibodies against human epidermal growth factors. In: *Growth and differentiation of cells in defined environment*. Murakami *et al.* (eds). Springer-Verlag: Berlin.